# Pregnancy Complications in Uterine Anomalies—A Pilot Study

**DOI:** 10.3390/jcm15082827

**Published:** 2026-04-08

**Authors:** Claudiu Voic, Melinda Ildiko Mitranovici, Septimiu Voidazan, Cezara Maria Mureşan, Elena Silvia Bernad

**Affiliations:** 1 Doctoral School, Victor Babes University of Medicine and Pharmacy from Timișoara, 300041 Timisoara, Romania; claudiu.voic@umft.ro; 2 Department of Obstetrics and Gynecology, Emergency County Hospital Hunedoara, 14 Victoriei Street, 331057 Hunedoara, Romania; 3 Department of Epidemiology, “George Emil Palade” University of Medicine, Pharmacy, Sciences and Technology, 540142 Targu Mures, Romania; septimiu.voidazan@umfst.ro; 4 Department of Obstetrics and Gynecology, Faculty of Medicine, Victor Babes University of Medicine and Pharmacy, 300041 Timisoara, Romania; muresan.maria@umft.ro (C.M.M.); bernad.elena@umft.ro (E.S.B.); 5 Center for Laparoscopy, Laparoscopic Surgery and In Vitro Fertilization, Faculty of Medicine, Victor Babes University of Medicine and Pharmacy, 300041 Timisoara, Romania; 6 Clinic of Obstetrics and Gynecology, Laparoscopy, In Vitro Fertilization and Embryotransfer Research Center, Pius Brinzeu County Clinical Emergency Hospital, 300723 Timisoara, Romania; 7 Center for Neuropsychology and Behavioral Medicine, Victor Babes University of Medicine and Pharmacy, 300041 Timisoara, Romania

**Keywords:** uterine anomalies, pregnancy complications, limit of viability, prenatal counseling, mental health

## Abstract

Uterine malformation represents a rare disease with a prevalence of up to 7% of the general population. **Background/Objectives**: Higher pregnancy complication rates have been reported in the literature; thus, in our study, we aimed to examine not only the obstetric complications encountered but also the psychological interventions and multidisciplinary approaches for parental counseling in our department in the context of preterm birth at the limit of viability. **Methods**: A retrospective pilot study was conducted on all the women in our department between 2010 and 2017 with congenital uterine malformations associated with infertility or pregnancy. In the study group, we included women with AUCs (*n* = 26), while the control group included pregnant women with normal uteri (*n* = 25) (total: *n* = 51), and then pregnancy complications were investigated. **Results**: Highly significant pregnancy complications were observed in the study group, the most important being preterm birth (*p* = 0.003) in comparison with the control group. Out of 26 patients with AUCs, only 14 gave birth to a live fetus compared to 22 out of the 25 with normal pregnancies, meaning that failure to give birth to a live newborn statistically significantly increased among the former group (*p* = 0.004). In terms of birth weight (*p* = 0.0001), Apgar score (*p* = 0.029) and intensive care unit admission (*p* = 0.0001), we observed significant differences between the newborns in the study group versus controls, with an impact on mental state that required psychological support. **Conclusions**: A clear correlation was observed in our study between uterine congenital malformations and pregnancy complications. The most common pregnancy outcome was premature delivery, with statistical significance. In addition, higher neonate admissions to the intensive care unit associated with lower Apgar scores were encountered compared with normal pregnancies. Appropriate parental counseling by obstetricians, neonatologists and psychologists could enhance pregnancy outcomes.

## 1. Introduction

The uterine malformation prevalence is 7% of the general population [1,2,3,4], including pathologies that may be affected by inaccurate diagnostic techniques [5]. Higher associated rates of pregnancy complications have been reported in the literature, such as miscarriage, preterm delivery, miscarriage, fetal death, ectopic pregnancy, malpresentation, intrauterine growth restriction or other obstetric complications [4,5,6,7,8,9,10]. High-risk pregnancy is associated with emotional stress, and thus professional support contributes to these pregnant women’s emotional well-being [11].

Many classifications have been proposed for uterine malformations, with the most accurate being that of the American Society of Reproductive Medicine [1,12]. In addition, the European Society of Human Reproduction and Embryology/European Society for Gynecological Endoscopy (ESHRE/ESGE) classification is relevant and has increased clinical impact [2]. Five main classes are included in this classification system: dysmorphic T-shaped uteri; septate uteri; uterus didelphys and bicornuate uteri; unicorporeal or unicornuate uteri; and aplastic uteri. In this classification, unclassified is class 6, while normal uteri are class 0 [2,13].

Different techniques are used to diagnose congenital uterine anomalies (CUAs): the most prominent is ultrasound, including 3D ultrasound (3D US), followed by hysterosalpingography (HSG), magnetic resonance imaging (MRI), hysteroscopy, and laparoscopy [2]. According to most studies, uterine malformations are associated with poor obstetric outcomes [4]. Surgical strategies have been employed to improve reproductive outcomes, but their effectiveness has not yet been demonstrated [1,8].

CUAs have been relatively understudied due to their rarity [5,6], meaning that their association with heavy mental pressure is poorly evaluated. Parental stress related to female infertility, poor obstetric outcomes, and premature infant hospitalization is a significant concern [14]. Approximately 64% of parents are affected by the inability to achieve spontaneous conception [14,15], a situation that also affects their quality of life, with adverse social consequences such as marriage breakdown. Targeted psychological interventions are required to improve their quality of life [16].

In this context, through our study, we aim to examine not only the obstetric complications encountered but also the psychological interventions and multidisciplinary approaches for parental counseling in our department. Our intention is to raise awareness of the importance of uterine malformation and the necessity of psychological interventions among both professionals and patients. Further studies are needed in order to find new and effective methods to improve fertility and pregnancy outcomes.

## 2. Materials and Methods

We conducted a retrospective pilot study on all women with congenital uterine malformations in our department between 2010 and 2017. The inclusion criteria were women with uterine abnormalities such as Müllerian agenesia, bicornuate uterus, unicornuate uterus, uterus didelphys, and septate uterus, determined according to the American College of Obstetricians and Gynecologists (ACOG) classification [1,12] as being associated with infertility or pregnancy. Infertility was defined according to the World Health Organization (WHO) criteria: patients with difficulty achieving a pregnancy after 12 months [12]. Exclusion criteria were as follows: genital malformations encountered outside of the context of infertility or pregnancy, multiple pregnancies, or pregnancies with fetal anomalies; complex malformations were also excluded. In the study group, we included women with CUAs (*n* = 26). Pregnancies in case of normal uteri were included in the reference group (*n* = 25). We included a total of 51 women in our study. We recorded data about maternal obstetric and demographic characteristics.

The description of the malformations was obtained based on the ACOG recommendation [1,12] using 3D transvaginal ultrasonography, hysteroscopy and laparoscopy. Surgical procedures were the treatment tool used for this specific purpose, and also to confirm the diagnosis suggested by ultrasound. We evaluated the maternal evolution, as well as the short-term neonatal outcomes, such as birth weight, sex, Apgar score and intensive care unit admission, and then we compared the two groups.

Signed informed consent was obtained from all patients upon admission to our department. The Ethics Committee of the Clinical Studies of Emergency County Hospital Al Simionescu Hunedoara approved the research protocol and the publication of the articles (ethical approval number: 16552/04.11.2024).

### Statistical Analysis

The normality distribution of numerical variables was assessed using the Kolmogorov–Smirnov test. Numerical data are expressed as means (standard deviation) or as medians (interquartile range) for normally and non-normally distributed data, respectively.

The Chi-squared test was used to compare categorical variables with the overall *p*-values provided in the tables. The level of statistical significance was set at *p* < 0.05, and all statistical tests were performed with SPSS, version 23.0.0 (SPSS Inc., Chicago, IL, USA).

## 3. Results

We have divided this section into several subsections based on the different factors analyzed.

### 3.1. Demographic Data and Diagnostic Tools

Comparing the study group of patients with uterine malformations in the context of pregnancy (*n* = 26) and the control group of women with normal pregnancies (*n =* 25), demographic data show non-statistically significant differences.

We present the types of malformations and diagnostic methods in the table below; 3D transvaginal ultrasound was the main technique, and we applied the Chi-squared test. Hysteroscopy and laparoscopy were, until recently, the gold standard in diagnostic procedures for AUCs. Even if these techniques are invasive, they allow for concurrent surgical correction. While a normal uterus does not require additional diagnostic methods, besides ultrasound, in case of AUCs, we sometimes need other investigations. In some of our cases, a combination of these techniques was applied (Table 1).

### 3.2. Types of Pregnancy-Related Complications Encountered

The most common pregnancy-related complication found in women with CUAs is preterm birth. We identified seven miscarriages, three infertility cases and eight premature births among the twenty-six women in our study group. These complications have a significantly severe impact on women with uterine anomalies (*p* = 0.003) (Table 2).

Then, we analyzed the relation between the types of malformations and pregnancy complications encountered and observed that the most frequent malformation was a subseptate uterus (11 from 26), associated with miscarriage in three cases, preterm birth, and placenta praevia in three cases. Mullerian agenesis led to infertility in 100% of instances. A miscarriage with serious bleeding was obtained in a septate uterus (Table 3).

The results in the statistics were included separately, but since in some cases two or three concurrent results were encountered, due to a potential causal relationship, we chose to display them this way precisely for clearer evidence; otherwise, the total number of outcomes is greater precisely through cumulation.

Significantly poorer pregnancy outcomes were obtained in the malformation group compared to the control group, as shown in Table 2 (*p* = 0.003). The most severe outcomes, such as infertility, were encountered in Müllerian agenesia or a bicornuate uterus. In addition, ectopic pregnancy followed by hysterectomy was encountered in the case of a unicornuate uterus with a rudimentary horn, while pregnancies in cases of a subseptate uterus and uterus didelphys were associated with the best outcomes in our study.

### 3.3. Types of Deliveries and Interventions

Out of 26 patients with CUAs, only 14 gave birth to a live fetus, compared to 22 out of 25 of those with normal pregnancies, meaning that the failure to give birth to a live newborn was statistically significantly increased (*p* = 0.004). The difference between groups regarding cesarean section and vaginal delivery was not significantly affected (Table 4).

Regarding types of surgical interventions used in this study, the most common procedure was cerclage in seven cases (26.9%); in 50% of CUAs, no procedure was applied (Table 5).

In our study, infertility was encountered in Müllerian agenesia along with bicornuate and septate uteri. Miscarriages were also associated with a bicornuate and septate uterus. Metroplasty was not successful in cases of a bicornuate uterus, while hysteroscopic septum resection was successful in only one case of a subseptate uterus, followed by premature birth. In one situation, a hysterectomy was used in the case of an ectopic pregnancy in the rudimentary uterine horn.

### 3.4. Neonatal Outcomes

Significant differences were encountered in the neonate outcomes in the two groups. We applied the Student test to compare the birth weight of newborns in the two groups, which was statistically significantly lower in patients with uterine anomalies (Table 6).

Apgar scores recorded at 1 min were also compared, with low scores reported in four cases in the study group, reaching statistical significance (*p* = 0.029) (Table 7).

Neonatal Intensive Care Unit (NICU) admission only occurred in the malformation group, and this difference was statistically significant (*p* = 0.001). The NICU admission was reported in cases of uterus didelphys, uterus bicornuate and uterus subseptate (Table 8).

### 3.5. Multidisciplinary Approach to Prenatal Parental Counseling

Considering the pregnancy complications associated with uterine anomalies, a multidisciplinary approach was necessary in a statistically significant proportion of cases (*p* = 0.002) (Table 9). The impact on mental state necessitated psychological support, especially in cases of infertility and premature infants born at the limit of viability. Prenatal counseling was chosen with the inclusion of a neonatologist to provide an accurate explanation of the infant’s evolution. In normal pregnancies, only an obstetrician or an obstetrician and neonatologist were necessary. Parental inclusion in therapeutic decision-making, however, was not statistically significant (Table 10).

## 4. Discussion

Uterine malformations are rare diseases associated with poor pregnancy outcomes [1,2,3,4]. As we have structured the results, in the discussions we focus on the aspects pursued: pregnancy outcomes in AUCS compared with normal uterus, diagnostic tools used in our study compared with the literature, surgical procedures, multidisciplinary approach and ethical concerns.

In our research, we observed the poorest obstetric outcomes in the case of Müllerian agenesia (infertility), followed by bicornuate uteri (infertility and miscarriage) (*p* = 0.003). Similar results were obtained by Pedro [4], wherein the bicornuate uterus groups were associated with pregnancy complications. This situation is linked with the underdiagnosis of congenital uterine anomalies (CUAs) [17]. According to Kang et al. (2024) [18], CUAs are associated with poor obstetrical outcomes such as higher early miscarriage rates and lower live birth rates [18]. However, women with CUAs have a significantly higher risk of developing pregnancy complications, including infertility, compared to women with a normal uterus [13]. Lin et al. (2004) [19] found in their research that the poorest reproductive outcomes were obtained in women with unicornuate uteri, while an arcuate uterus, which is a variant of a normal uterus, is not associated with pregnancy complications. In case of a septate uterus, an increased miscarriage rate was detected [19]. Our findings are similar to the literature. Analyzing the relation between malformation types and pregnancy outcomes, the most frequent malformation encountered was a subseptate uterus (11 of 26), associated with miscarriage in three cases, and placenta praevia, associated with preterm birth in three cases. In one case of a unicornuate uterus with a rudimentary horn, ectopic pregnancy was followed by hysterectomy. Only 14 out of 26 patients with CUAs gave birth to a live fetus compared to 22 out of 25 of those with normal pregnancies, meaning that the failure to give birth to a live newborn was statistically significantly increased (*p* = 0.004). However, any anatomical defects can lead to complications, while successful embryo implantation depends on a proper endometrial cavity [5]. The best outcomes were therefore obtained in pregnancies associated with a subseptate uterus and uterus didelphys, followed by preterm birth. Preterm birth was observed in uteri didelphys, unicornuate uteri, and subseptate uteri. Preterm birth was associated with other complications, for example, placenta praevia, breech presentation, or bleeding and also occurred alone. Unicornuate and didelphys uteri were also followed by miscarriages. However, according to De Angelis (2015), a successful pregnancy is possible [20], but didelphys uteri have a higher risk of preterm delivery [21] and IUGR [22]. In our study, the most important complication encountered in women with CUAs was premature delivery, even after proper management of such pregnancies, which is similar to data in the literature [21,22,23,24,25].

Uterine malformations remained underdiagnosed in our study, even if ultrasound demonstrated its efficacy. Confusion still exists in diagnosing such cases, especially between arcuate and subseptate uteri. Also, an ectopic pregnancy developed in the rudimentary horn of a woman with a unicornuate uterus was diagnosed only through laparotomy. This was corroborated by Li et al. (2019), who described a rupture of an ectopic first-trimester pregnancy developed in the rudimentary horn [26]. Mistakes occur when investigating women with CUAs, which may lead to pregnancy mismanagement [27]. CUME definitions for these conditions could help in diagnosing uterine malformations [17].

The successful diagnostic tool used in our study was 3D transvaginal ultrasonography; however, other techniques were used, such as hysteroscopy, hysterography, and laparoscopy. Surgical interventions were considered for treatment, with cerclage being the most common surgical procedure used successfully in our study, reaching statistical significance (*p* = 0.005). Cervical incompetence was not associated with any of these cases. Based on the findings in the literature, this procedure can be used most of the time with remarkable results [4,28]. However, the gold standard in AUCs diagnosis remains MRI [29,30]

Related to surgical interventions, in our study, the uterine cavity obtained after metroplasty was insufficient for the development of a normal pregnancy, and was followed by miscarriage. According to the literature, the rate of pregnancy can not be increased after metroplasty [4], and according to Wang et al. (2019), the metroplasty was followed by infertility, a procedure also used in a case of a complete uterine septum [31]. On the contrary, Ganti et al. (2024) suggested that hysteroscopic metroplasty can significantly improve pregnancy outcomes [29], while Shokeir (2004) performed hysteroscopic septoplasty in women with unexplained infertility [32]. In our research, hysteroscopic septum resection was successful in only one case of a subseptate uterus, followed by premature birth. Afterward, a high proportion of the affected women can conceive spontaneously [31]. According to Akhtar et al. (2020), surgical procedures used as treatment for CUAs are not recommended because of the high risk they pose compared to their potential benefits [30].

In our study, a rudimentary uterine horn was complicated by an ectopic pregnancy that ended with a hysterectomy. This was successfully diagnosed through ultrasound and confirmed after the surgical procedure.

Additionally, we analyzed neonatal outcomes in the pregnancies of women with CUAs compared with those in women with normal uteri. We encountered a low Apgar score (*p* = 0.029) and high NICU admissions of neonates delivered by pregnant women with CUAs, which were significantly higher than in the control group (*p* = 0.001). Birth weight was also statistically significantly lower in patients with uterine anomalies (*p* = 0.0001). Our findings are similar to those obtained in a case report by Vaz et al. (2017) [33]. Furthermore, Abe et al. reported severe cerebral palsy, a long-term neuro-developmental disorder, in a neonate after a pregnancy associated with a bicornuate uterus [34]. Müllerian defects could be considered a risk factor for poor pregnancy outcomes, associated with placental abruption, intrauterine growth restriction, preeclampsia, and preterm delivery with high perinatal mortality [29,33]. Abnormal placental cord insertion was another complication reported in cases of pregnancies complicated by a CUA, which is associated with poor pregnancy outcomes [30,35].

Proper management of these conditions depends on a universally accepted classification system, which is needed in order to find appropriate prevention strategies [28,36]. In the future, different diagnostic modalities will be necessary for precise classification [24,37], but until then, CUAs can be easily misdiagnosed. According to Akkus et al. (2024), over one year, anomalies did not receive prior diagnosis, being discovered during cesarean section in a tertiary center [38].

A multidisciplinary approach was necessary in a statistically significant proportion (*p* = 0.002) of patients in our research. Abhinaya et al. conducted a study (2024), finding that those with CUAs require proper counseling in the antenatal period and proper monitoring during labor [24,39]. According to Hendy, three-quarters of parents experienced high overall stress levels [14], while in our study, we identified an increased need for psychological support in association with a multidisciplinary approach, with a neonatologist being involved along with obstetricians. In cases of prematurity at the limit of viability, prenatal counseling should be introduced in clinical practice and adjusted to parental needs. Heterogeneity has been found among practitioners and trainees, and system-based hospital variation has been found [40].

Gestational age limits regarding fetal viability have been gradually reduced because of advances in neonatal care. In decision-making processes, obstetricians should employ neonatologists and psychologists in guiding families. Parents will face challenges because of the high chances of severe sequelae of premature infants [41,42]. However, a study was conducted to identify the preferences of adults with children born prematurely because of the ethical considerations regarding the future of these infants. The author emphasized that these adults prefer a periviability guideline that takes into consideration multiple prognostic factors [43]; the FIGO guideline explores the ethical complexities of this issue [44]. Other studies were employed to analyze who should make decisions on lifesaving treatment for extremely premature infants born at the limits of viability, including considering parents’ and healthcare professionals’ involvement [45]. A multidisciplinary approach to psychological support for parents confronting such dilemmas was also analyzed, and according to Hendy et al., even marital status and mental health were affected [14]. In our research, marital status was not affected by CUAs and pregnancy complications related to these pathologies.

The strength of our study lies in the possibility of appropriate diagnosis and surgical management in cases of pregnancy associated with a uterine malformation. This was a quick, inexpensive retrospective study that identified gaps in the current knowledge and helped us to design a future appropriate management strategy. Women with uterine malformations should benefit from a multidisciplinary approach and psychological counseling, and should be informed about the CUA’s impact on fertility and obstetric outcomes; however, we observed a lack of awareness of the problems associated with CUAs in both patients and gynecologists.

A major limitation of our study is that it is an observational retrospective study. It was conducted in a single center. However, we used 3D transvaginal ultrasound, which is currently the most effective method along with MRI, the achieved accuracies exceeding 90–97% [46]. The small sample size is another important limitation, but it is consistent with those in the literature, with malformations being rare in the general population according to ASRM [47]. Further studies are needed in a multicentric study using additional diagnostic tools. A genetic perspective should be useful.

## 5. Conclusions

In our research, we observed a clear correlation between uterine congenital malformations and pregnancy complications. The most common pregnancy outcome was premature delivery, reaching statistical significance. Lower Apgar scores and higher neonate admissions to the intensive care unit were also encountered compared with normal pregnancies. Optimal management should incorporate multidisciplinary approaches, with appropriate parental counseling conducted by obstetricians, neonatologists and psychologists. Accurate information based on good practice and valid classification systems, and an increased awareness of these pathologies, could increase the chances of therapy being adapted to the situation for future pregnant women. This pilot study opens new fields for further research.

## Figures and Tables

**Table 1:** Diagnostic technique uterine malformation crosstabulation.

*p* = 0.0001			Malformation		Total
			No	Yes	
diagnosis	hysteroscopy	Count	0	1	1
		% within Malformation	0.0%	3.8%	2.0%
	hysteroscopy and ultrasound	Count	0	1	1
		% within Malformation	0.0%	3.8%	2.0%
	hysterography/hysteroscopy	Count	0	3	3
		% within Malformation	0.0%	11.5%	5.9%
	hysteroscopy	Count	0	2	2
		% within Malformation	0.0%	7.7%	3.9%
	laparoscopy	Count	0	1	1
		% within Malformation	0.0%	3.8%	2.0%
	laparotomy	Count	0	1	1
		% within Malformation	0.0%	3.8%	2.0%
	3D transvaginal ultrasound (3D-TVUS)	Count	25	7	32
		% within Malformation	100.0%	26.9%	62.7%
	3D-TVUS/hysterography	Count	0	4	4
		% within Malformation	0.0%	15.4%	7.8%
	3D-TVUS/hysterography/hysteroscopy	Count	0	1	1
		% within Malformation	0.0%	3.8%	2.0%
	3D-TVUS/laparoscopy	Count	0	3	3
		% within Malformation	0.0%	11.5%	5.9%
	3D-TVUS/MRI	Count	0	2	2
		% within Malformation	0.0%	7.7%	3.9%
Total		Count	25	26	51
		% within Malformation	100.0%	100.0%	100.0%

3D-TVUS = three-dimensional transvaginal ultrasound, MRI magnetic resonance imaging.

**Table 2:** Comparison between pregnancy complications in the study group with malformations and those with normal pregnancies.

*p* = 0.003 Significance			Malformation		Total
			No	Yes	
pregnancy complications	breech presentation	Count	1	1	2
		% within Malformation	4.0%	3.8%	3.9%
	breech presentation and placenta praevia	Count	0	1	1
		% within Malformation	0.0%	3.8%	2.0%
	breech presentation, and preterm birth	Count	0	2	2
		% within Malformation	0.0%	7.7%	3.9%
	placental abruption	Count	1	0	1
		% within Malformation	4.0%	0.0%	2.0%
	dystocic pelvis	Count	1	0	1
		% within Malformation	4.0%	0.0%	2.0%
	ectopic pregnancy in a rudimentary horn	Count	0	1	1
		% within Malformation	0.0%	3.8%	2.0%
	infertility	Count	0	3	3
		% within Malformation	0.0%	11.5%	5.9%
	IUGR	Count	1	0	1
		% within Malformation	4.0%	0.0%	2.0%
	miscarriage	Count	3	6	9
		% within Malformation	12.0%	23.1%	17.6%
	miscarriage with important bleeding	Count	0	1	1
		% within Malformation	0.0%	3.8%	2.0%
	no	Count	13	3	16
		% within Malformation	52.0%	11.5%	31.4%
	oligoamnios	Count	1	0	1
		% within Malformation	4.0%	0.0%	2.0%
	preeclampsia	Count	2	0	2
		% within Malformation	8.0%	0.0%	3.9%
	preterm birth	Count	1	5	6
		% within Malformation	4.0%	19.2%	11.8%
	preterm birth and placenta praevia	Count	1	3	4
		% within Malformation	4.0%	11.5%	7.8%
Total		Count	25	26	51
		% within Malformation	100.0%	100.0%	100.0%

We applied the Chi-squared test, and it had a statistically significant association of *p* < 0.05.

**Table 3:** Pregnancy complications and genital uterine malformations.

*p* = 0.12			Genit. Malf.								Total
			Mullerian Agenesia	*n*	Septate Uterus	Subseptate Uterus	Unicornuate Uterus	Unicornuate Uterus with Rudimentary Horn	Uterus Didelphys	Uterus Bicornuate	
pregnancy complications	breech presentation	Count	0	1	0	1	0	0	0	0	2
		% within genit. malf.	0.0%	4.0%	0.0%	9.1%	0.0%	0.0%	0.0%	0.0%	3.9%
	breech presentation and placenta praevia	Count	0	0	0	0	1	0	0	0	1
		% within genit. malf.	0.0%	0.0%	0.0%	0.0%	25.0%	0.0%	0.0%	0.0%	2.0%
	breech presentation and preterm birth	Count	0	0	0	0	1	0	1	0	2
		% within genit. malf.	0.0%	0.0%	0.0%	0.0%	25.0%	0.0%	50.0%	0.0%	3.9%
	Placental abruption	Count	0	1	0	0	0	0	0	0	1
		% within genit. malf.	0.0%	4.0%	0.0%	0.0%	0.0%	0.0%	0.0%	0.0%	2.0%
	dystocic pelvis	Count	0	1	0	0	0	0	0	0	1
		% within genit. malf.	0.0%	4.0%	0.0%	0.0%	0.0%	0.0%	0.0%	0.0%	2.0%
	ectopic pregnancy in a rudimentary horn	Count	0	0	0	0	0	1	0	0	1
		% within genit. malf.	0.0%	0.0%	0.0%	0.0%	0.0%	50.0%	0.0%	0.0%	2.0%
	infertility	Count	1	0	1	0	0	0	0	1	3
		% within genit. malf.	100.0%	0.0%	33.3%	0.0%	0.0%	0.0%	0.0%	33.3%	5.9%
	Intrauterine growth restriction	Count	0	1	0	0	0	0	0	0	1
		% within genit. malf.	0.0%	4.0%	0.0%	0.0%	0.0%	0.0%	0.0%	0.0%	2.0%
	miscarriage	Count	0	3	1	3	0	1	0	1	9
		% within genit. malf.	0.0%	12.0%	33.3%	27.3%	0.0%	50.0%	0.0%	33.3%	17.6%
	miscarriage with important bleeding	Count	0	0	1	0	0	0	0	0	1
		% within genit. malf.	0.0%	0.0%	33.3%	0.0%	0.0%	0.0%	0.0%	0.0%	2.0%
	no	Count	0	13	0	2	1	0	0	0	16
		% within genit. malf.	0.0%	52.0%	0.0%	18.2%	25.0%	0.0%	0.0%	0.0%	31.4%
	oligoamnios	Count	0	1	0	0	0	0	0	0	1
		% within genit. malf.	0.0%	4.0%	0.0%	0.0%	0.0%	0.0%	0.0%	0.0%	2.0%
	preeclampsia	Count	0	2	0	0	0	0	0	0	2
		% within genit. malf.	0.0%	8.0%	0.0%	0.0%	0.0%	0.0%	0.0%	0.0%	3.9%
	preterm birth	Count	0	1	0	2	1	0	1	1	6
		% within genit. malf.	0.0%	4.0%	0.0%	18.2%	25.0%	0.0%	50.0%	33.3%	11.8%
	preterm birth and placenta praevia	Count	0	1	0	3	0	0	0	0	4
		% within genit. malf.	0.0%	4.0%	0.0%	27.3%	0.0%	0.0%	0.0%	0.0%	7.8%
Total		Count	1	25	3	11	4	2	2	3	51
		% within genit. malf.	100.0%	100.0%	100.0%	100.0%	100.0%	100.0%	100.0%	100.0%	100.0%

**Table 4:** Comparison: failure to deliver a live newborn with cesarean versus vaginal birth.

*p* = 0.004		Genit. Malf.								Total
		Mullerian Agenesia	*n*	Septate Uterus	Subseptate Uterus	Unicornuate Uterus	Unicornuate Uterus with Rudimentary Horn	Uterus Didelphys	Uterus Bicornuate	
Failure to give birth to a live newborn	Count	1	3	3	3	1	2	0	2	15
	% within genit. malf.	100.0%	12.0%	100.0%	27.3%	25.0%	100.0%	0.0%	66.7%	29.4%
C	Count	0	8	0	5	2	0	2	1	18
	% within genit. malf.	0.0%	32.0%	0.0%	45.5%	50.0%	0.0%	100.0%	33.3%	35.3%
V	Count	0	14	0	3	1	0	0	0	18
	% within genit. malf.	0.0%	56.0%	0.0%	27.3%	25.0%	0.0%	0.0%	0.0%	35.3%
Total	Count	1	25	3	11	4	2	2	3	51
	% within genit. malf.	100.0%	100.0%	100.0%	100.0%	100.0%	100.0%	100.0%	100.0%	100.0%

C = cesarean section; V = vaginal delivery.

**Table 5:** Surgical interventions required.

*p* = 0.005 Significance			Malformation		Total
			No	Yes	
intervention	a. Necessary intervention for failed pregnancy: curettage	Count	0	1	1
		% within Malformation	0.0%	3.8%	2.0%
	a. Necessary intervention for failed pregnancy: hysterectomy	Count	0	2	2
		% within Malformation	0.0%	7.6%	4.0%
	b. Intervention to improve fertility: hysteroscopic resection	Count	0	1	1
		% within Malformation	0.0%	3.8%	2.0%
	b. Intervention to improve fertility: hysteroscopic resection followed by cerclage	Count	0	1	1
		% within Malformation	0.0%	3.8%	2.0%
	b. Intervention to improve fertility: metroplasty	Count	0	1	1
		% within Malformation	0.0%	3.8%	2.0%
	c. Intervention during pregnancy to improve outcome: cerclage	Count	1	7	8
		% within Malformation	4.0%	26.9%	15.7%
	d. no intervention	Count	24	13	37
		% within Malformation	96.0%	50.0%	72.5%
Total		Count	25	26	51
		% within Malformation	100.0%	100.0%	100.0%

**Table 6:** Differences between the birth weights of newborns in the malformation group and the normal uterus group.

	Malformations	Normal Uterus	Mean	Std. Deviation	*p*-Value
birth weight	yes	14	2243.57	540.322	0.0001
	no	22	2975.91	460.752	

The student’s test was used; the mean birth weight and standard deviation were reported.

**Table 7:** APGAR scores recorded at 1 min in newborns from pregnancies with malformation versus the normal uterus groups.

*p* = 0.029 Significance			Malformation		Total
			No	Yes	
APGAR score at 1 min	5	Count	0	2	2
		% within Malformation	0.0%	14.3%	5.6%
	6	Count	1	2	3
		% within Malformation	4.5%	14.3%	8.3%
	7	Count	1	0	1
		% within Malformation	4.5%	0.0%	2.8%
	8	Count	3	2	5
		% within Malformation	13.6%	14.3%	13.9%
	9	Count	8	8	16
		% within Malformation	36.4%	57.1%	44.4%
	10	Count	9	0	9
		% within Malformation	40.9%	0.0%	25.0%
Total		Count	22	14	36
		% within Malformation	100.0%	100.0%	100.0%

**Table 8:** NICU admission in the malformation group compared to the control group.

*p* = 0.0001 Significance			Malformation		Total
			No	Yes	
NICU		Count	3	12	15
		% within Malformation	12.0%	46.2%	29.4%
	no	Count	22	11	33
		% within Malformation	88.0%	42.3%	64.7%
	yes	Count	0	3	3
		% within Malformation	0.0%	11.5%	5.9%
Total		Count	25	26	51
		% within Malformation	100.0%	100.0%	100.0%

**Table 9:** Multidisciplinary approach involving obstetrician/neonatologist/psychologist.

*p* = 0.002			Malformation		Total
			No	Yes	
multidisciplinary approach: O/N/P	no	Count	19	9	28
		% within Malformation	76.0%	34.6%	54.9%
	O, N	Count	5	6	11
		% within Malformation	20.0%	23.1%	21.6%
	O, N, P	Count	1	5	6
		% within Malformation	4.0%	19.2%	11.8%
	O, P	Count	0	6	6
		% within Malformation	0.0%	23.1%	11.8%
Total		Count	25	26	51
		% within Malformation	100.0%	100.0%	100.0%

O = obstetrician; N = neonatologist; P = psychologist.

**Table 10:** Parents’ involvement in treatment decision-making.

*p* = 0.75 Significance			Malformation		Total
			No	Yes	
Parents’ involvement	no	Count	3	4	7
		% within Malformation	12.0%	15.4%	13.7%
	yes	Count	22	22	44
		% within Malformation	88.0%	84.6%	86.3%
Total		Count	25	26	51
		% within Malformation	100.0%	100.0%	100.0%

## Data Availability

Raw data are available in the Al Simionescu County Hospital registers.

## Abbreviations

The following abbreviations are used in this manuscript:

ESHRE	European Society of Human Reproduction and Embryology
ESGE	European Society for Gynecological Endoscopy
CUA	congenital uterine anomaly
HSG	hysterosalpingography
MRI	magnetic resonance imaging
ACOG	American College of Obstetricians and Gynecologists
WHO	World Health Organization
NICU	Neonatal Intensive Care Unit
IUGR	intrauterine growth restriction

## References

[1] Bhagavath B., Greiner E., Griffiths K.M., Winter T., Alur-Gupta S., Richardson C., Lindheim S.R. (2017). Uterine malformations: An update of diagnosis, management, and outcomes. Obstet. Gynecol. Surv..

[2] Ludwin A., Ludwin I., Coelho Neto M.A., Nastri C.O., Bhagavath B., Lindheim S.R., Martins W.P. (2019). Septate uterus according to ESHRE/ESGE, ASRM and CUME definitions: Association with infertility and miscarriage, cost and warnings for women and healthcare systems. Ultrasound Obstet. Gynecol..

[3] Passos I.D.M.P., Britto R.L. (2020). Diagnosis and treatment of müllerian malformations. Taiwan. J. Obstet. Gynecol..

[4] Acién P. (1993). Reproductive performance of women with uterine malformations. Hum. Reprod..

[5] Kim M.A., Kim H.S., Kim Y.H. (2021). Reproductive, obstetric and neonatal outcomes in women with congenital uterine anomalies: A systematic review and meta-analysis. J. Clin. Med..

[6] Mandelbaum R.S., Anderson Z.S., Masjedi A.D., Violette C.J., McGough A.M., Doody K.A., Guner J.Z., Quinn M.M., Paulson R.J., Ouzounian J.G. (2024). Obstetric outcomes of women with congenital uterine anomalies in the United States. Am. J. Obstet. Gynecol. MFM.

[7] Naeh A., Sigal E., Barda S., Hallak M., Gabbay-Benziv R. (2022). The association between congenital uterine anomalies and perinatal outcomes–does type of defect matters?. J. Matern.-Fetal Neonatal Med..

[8] Gliozheni O., Gliozheni E. (2021). Congenital uterine anomalies: Impact on perinatal outcomes. Orion.

[9] Solanki K., Kochar S., Poonia L. (2020). Case series on obstetrical outcomes in patient with uterine malformations. Int. J. Reprod. Contracept. Obstet. Gynecol..

[10] Bulletti C., Simon C. (2019). Bioengineered uterus: A path toward ectogenesis. Fertil. Steril..

[11] Kozel E., Barnoy S., Itzhaki M. (2022). Emotion management of women at risk for premature birth: The association with optimism and social support. Appl. Nurs. Res..

[12] Goyal L.D., Dhaliwal B., Singh P., Ganjoo S., Goyal V. (2020). Management of mullerian development anomalies: 9 years’ experience of a tertiary care center. Gynecol. Minim. Invasive Ther..

[13] Al-Bdairi A.A., Al-Hindy H.A.A.M., Rahmatullah W.S., Alshukri W.S.M. (2024). Impact of congenital uterine anomalies on ectopic pregnancy: A cross-sectional observational study of 510 cases. Med. J. Babylon.

[14] Hendy A., El-Sayed S., Bakry S., Mohammed S.M., Mohamed H., Abdelkawy A., Hassani R., A Abouelela M., Sayed S. (2024). The stress levels of premature infants’ parents and related factors in NICU. SAGE Open Nurs..

[15] Burgio S., Polizzi C., Buzzaccarini G., Laganà A.S., Gullo G., Perricone G., Perino A., Cucinella G., Alesi M. (2022). Psychological variables in medically assisted reproduction: A systematic review. Menopause Rev./Przegląd Menopauzalny.

[16] Xie Y., Ren Y., Niu C., Zheng Y., Yu P., Li L. (2023). The impact of stigma on mental health and quality of life of infertile women: A systematic review. Front. Psychol..

[17] Ludwin A., Martins W.P., Nastri C.O., Ludwin I., Neto M.A.C., Leitão V.M., Acién M., Alcazar J.L., Benacerraf B., Condous G. (2018). Congenital Uterine Malformation by Experts (CUME): Better criteria for distinguishing between normal/arcuate and septate uterus?. Ultrasound Obstet. Gynecol..

[18] Kang J., Qiao J. (2024). Impact of congenital uterine anomalies on reproductive outcomes of IVF/ICSI-embryo transfer: A retrospective study. Eur. J. Med. Res..

[19] Lin P.C. (2004). Reproductive outcomes in women with uterine anomalies. J. Women’s Health.

[20] De Angelis C., Caserta D. (2015). Pregnancy outcome in women with uterine anomalies. Female Genital Tract Congenital Malformations: Classification, Diagnosis and Management.

[21] Shokeir T., Abdelshaheed M., El-Shafie M., Sherif L., Badawy A. (2011). Determinants of fertility and reproductive success after hysteroscopic septoplasty for women with unexplained primary infertility: A prospective analysis of 88 cases. Eur. J. Obstet. Gynecol. Reprod. Biol..

[22] Sánchez-Santiuste M., Ríos M., Calles L., Cuesta R.D.L., Engels V., Pereira A., Pérez-Medina T. (2020). Dysmorphic uteri: Obstetric results after hysteroscopic office metroplasty in infertile and recurrent pregnancy loss patients. A prospective observational study. J. Clin. Med..

[23] Violette C.J., Mandelbaum R.S., Doody K.J., Guner J.Z., Quinn M.M., Ho J.R., Ouzounian J.G., Paulson R.J., Matsuo K. (2022). Obstetric outcomes in women with congenital uterine anomalies: A big data approach. Fertil. Steril..

[24] Gandelsman E., Grin L., Wainstock T., Berkovitz Shperling R., Scherbina E., Saar-Ryss B. (2025). Risk of adverse pregnancy outcomes after abnormal hysterosalpingography. Hum. Fertil..

[25] Yoshihara T., Okuda Y., Yoshino O. (2024). Association of congenital uterine anomaly with abnormal placental cord insertion and adverse pregnancy complications: A retrospective cohort study. J. Matern.-Fetal Neonatal Med..

[26] Li X., Peng P., Liu X., Chen W., Liu J., Yang J., Bian X. (2019). The pregnancy outcomes of patients with rudimentary uterine horn: A 30-year experience. PLoS ONE.

[27] Buicu C.F., Mitranovici M.I., Dumitrascu Biris D., Craina M., Bernad E.S. (2025). Birth Outcomes in Pregnancies with Uterine Malformations: A Single-Center Retrospective Study. J. Clin. Med..

[28] Gaily A.S., Abdulaal N.A., Alzahrani A., Gaily A., Abdulaal N.A. (2024). A full-term pregnancy in a patient with uterus didelphys. Cureus.

[29] Ganti S., Arogyaswamy P., Srinivasan J., Archunan P.A., Srinivasan J., Aarthy P. (2024). Maternofetal Outcomes in Women with Congenital Uterine Anomalies. Cureus.

[30] Akhtar M.A., Saravelos S.H., Li T.C., Jayaprakasan K. (2020). Royal College of Obstetricians and Gynaecologists. Reproductive implications and management of congenital uterine anomalies: Scientific impact paper No. 62 November 2019. BJOG.

[31] Wang X., Hou H., Yu Q. (2019). Fertility and pregnancy outcomes following hysteroscopic metroplasty of different sized uterine septa: A retrospective cohort study protocol. Medicine.

[32] Shokeir T.A., Shalan H.M., El-Shafei M.M. (2004). Combined diagnostic approach of laparoscopy and hysteroscopy in the evaluation of female infertility: Results of 612 patients. J. Obstet. Gynaecol. Res..

[33] Vaz S.A., Dotters-Katz S.K., Kuller J.A. (2017). Diagnosis and management of congenital uterine anomalies in pregnancy. Obstet. Gynecol. Surv..

[34] Abe J., Nasu T., Noro A., Tsubaki J. (2024). An unusual case of severe asphyxia with the fetal position unexpectedly inverted in a malformed uterus: A case report. J. Med. Case Rep..

[35] Yoshihara T., Okuda Y., Yoshino O. (2025). Diagnosis of arcuate uterus using three-dimensional transvaginal ultrasound and investigation of its association with perinatal complications. Int. J. Gynecol. Obstet..

[36] Silsby Z.O., Rhodes S., Kaelber D.C., Sheyn D., Lappen J.R. (2024). Adverse pregnancy outcomes in patients with congenital uterine anomalies: Evaluation of a large population database. Am. J. Obstet. Gynecol..

[37] Moraru L., Mitranovici M.I., Chiorean D.M., Moraru R., Caravia L., Tiron A.T., Cotoi O.S. (2023). Adenomyosis and its possible malignancy: A review of the literature. Diagnostics.

[38] Akkuş Z.C., Celik O.Y., Karadeniz R.S. (2024). How Often Do We Discover an Abnormality of The Uterus at Delivery? Single Center Experience. Türk Kadın Sağlığı Neonatoloji Derg..

[39] Abhinaya M., Rani B.U., Adilakshmi V., Babu N.B. (2024). A Study of Mullerian Anomalies in Pregnancy: Case Series in a Tertiary Care Centre. Int. J. Med. Public Health.

[40] De Proost L., Geurtzen R., M’hamdi H.I., Reiss I.I., Steegers E.E., Verweij E.J. (2022). Prenatal counseling for extreme prematurity at the limit of viability: A scoping review. Patient Educ. Couns..

[41] Martone P., Molas A., Marre D. (2025). Between ‘fetal viability’ and the ‘viability of families’: Decision-making for extremely premature infants in Spain. Soc. Sci. Med..

[42] Kornhauser Cerar L., Lucovnik M. (2023). Ethical dilemmas in neonatal care at the limit of viability. Children.

[43] De Proost L., de Boer A., Reiss I.K.M., Steegers E.A.P., Verhagen A.A.E., Hogeveen M., Geurtzen R., Verweij E.J. (2023). Adults born prematurely prefer a periviability guideline that considers multiple prognostic factors beyond gestational age. Acta Paediatr..

[44] Vidaeff A.C., Capito L., Gupte S., Antsaklis A., FIGO Committee on the Ethical Aspects of Human Reproduction and Women’s Health (2024). The ethics and practice of perinatal care at the limit of viability: FIGO recommendations. Int. J. Gynecol. Obstet..

[45] Kelly J., Welch E. (2018). Ethical decision-making regarding infant viability: A discussion. Nurs. Ethics.

[46] Jayaprakasan K., Ojha K. (2022). Diagnosis of congenital uterine abnormalities: Practical considerations. J. Clin. Med..

[47] Ludwin A., Tudorache S., Martins W.P. (2022). ASRM Mullerian Anomalies Classification 2021: A critical review. Ultrasound Obstet. Gynecol..

